# Three new species of soil-inhabiting *Trichoderma* from southwest China

**DOI:** 10.3897/mycokeys.44.30295

**Published:** 2018-12-14

**Authors:** Min Qiao, Xing Du, Zhe Zhang, JianPing Xu, ZeFen Yu

**Affiliations:** 1 School of Life Sciences, Yunnan University, No. 2 North, Kunming, Yunnan, 650091, P. R. China; 2 Laboratory for Conservation and Utilization of Bio-resources, Key Laboratory for Microbial Resources of the Ministry of Education, Yunnan University, Kunming, Yunnan, 650091, P. R. China; 3 Department of Biology, McMaster University, Hamilton, Ontario, L8S 4K1, Canada

**Keywords:** Rhizospheric fungi, diversity, Hypocreales, taxonomy

## Abstract

Fungi in the genus *Trichoderma* are widely distributed in China, including in Yunnan province. In this study, we report three new soil-inhabiting species in *Trichoderma*, named as *T.kunmingense*, *T.speciosum* and *T.zeloharzianum*. Their colony and mycelial morphology, including features of asexual states, were described. For each species, their DNA sequences were obtained from three loci, the internal transcribed spacer (ITS) regions of the ribosomal DNA, the translation elongation factor 1-α encoding gene (tef1) and the gene encoding the second largest nuclear RNA polymerase subunit (rpb2). Our analyses indicated that the three new species showed consistent divergence amongst each other and from other known and closely related species. Amongst the three, *T.speciosum* and *T.kunmingense* belong to the Viride Clade. Specifically, *T.speciosum* is related to three species – *T.hispanicum*, *T.samuelsii* and *T.junci* and is characterised by tree-like conidiophores, generally paired branches, curved terminal branches, spindly to fusiform phialides and subglobose to globose conidia. In contrast, *T.kunmingense* morphologically resembles *T.asperellum* and *T.yunnanense* and is distinguished by its pyramidal conidiophores, ampulliform to tapered phialides, discrete branches and ovoidal, occasionally ellipsoid, smooth-walled conidia. The third new species, *T.zeloharzianum*, is a new member of the Harzianum Clade and is closely associated with *T.harzianum*, *T.lixii* and *T.simmonsii* but distinguished from them by having smaller, subglobose to globose, thin-walled conidia.

## Introduction

The genus *Trichoderma* Pers. (Ascomycota, Sordariomycete, Hypocreales, teleomorph *Hypocrea* Fr.) is cosmopolitan, often existing as saprophytes in a diversity of ecosystems, such as agricultural fields, prairies, forests and salt marshes (Gond et al. 2007, Verma et al. 2007, Gazis and Chaverri 2010). Though rarely, they are also found in deserts and freshwater ecosystems. In some woody plants, they are the most abundant endophytes. In addition, a few species of *Trichoderma* are effective in attacking or inhibiting other fungi through their secondary metabolites and these fungi have been exploited as potential biocontrol agents against plant pathogens (Degenkolb et al. 2008, Cheng et al. 2012, Lopes et al. 2012, Mukherjee et al. 2013). A few *Trichoderma* species are crop pathogens and can produce toxins to spoil food. For example, *T.aggressivum* can cause significant crop loss to mushroom production (Oda et al. 2009, Schuster and Schmoll 2010, Kim et al. 2012, 2013).

As multilocus molecular phylogeny enables rapid and accurate identification of *Trichoderma* species, a significant number of *Trichoderma* species have been recently reported based on molecular phylogenetic evidence. Following the guidelines of the International Code of Nomenclature (ICN) for algae, fungi and plants (Melbourne Code, Art. 14.13), 254 names of *Trichoderma* species and two names of varieties in *Trichoderma* were accepted in 2015 (Bissett et al. 2015). Since then, 71 new *Trichoderma* species have been reported. Amongst these 71 species, 15 were described based on cultures from ascospores and the remaining 56 were based on asexual morphs in nature (Chen and Zhuang 2016, 2017a, b, c, Qin and Zhuang 2016a, b, c, d, 2017, Zhu et al. 2017a, b, du Plessis et al. 2018). Most of the new species were isolated from soil (Chen and Zhuang 2016, 2017a, b, c, du Plessis et al. 2018), rotten twigs, stems or barks (Qin and Zhuang 2016a, b, c, d, 2017, Zhu et al. 2017a, b). Several were found associated with the attine ants (Montoya et al. 2016) and on the surface of *Hypoxylonanthochroum* stroma (Sun et al. 2016).

China has an enormous fungal diversity. Amongst the 71 new *Trichoderma* species reported since 2015, 43 were from China (Chen and Zhuang 2017a,b,c, Qin and Zhuang 2017, Zhu et al. 2017a, b). Of these 43 species, 33 were from the soil of different regions (Chen and Zhuang 2017a, b, c), which shows that soil has a high *Trichoderma* diversity. In our survey of *Trichoderma* from soil, 180 *Trichoderma* strains were collected in southwest China and preserved in the Laboratory for Conservation and Utilization of Bioresources, Yunnan University (YMF) and China General Microbiological Culture Collection Center (CGMCC). Three new species were identified based on morphological features and DNA sequence data at three loci: the genes encoding RNA polymerase II subunit (rpb2) and translation elongation factor 1-α gene (tef1) and the internal transcribed spacer (ITS) regions of the nuclear ribosomal RNA gene cluster. Based on the DNA sequence information, we revealed their phylogenetic positions as belonging to the Viride Clade (two species) and the Harzianum Clade (one species).

## Materials and methods

### Isolates of strains

Soil samples were collected from Luliang and Kunming in Yunnan Province, southwest China. All the samples were stored at 4 °C before use. *Trichoderma* strains were obtained by serial dilutions (1,000 to 1,000,000 fold) and spread on to the surface of Rose Bengal agar with antibiotics (40 mg streptomycin, 30 mg ampicillin per litre) added in a 9-cm-diam. Petri dish, followed by incubation under 25 °C for 5 days. Representative colonies were picked up with a sterilised needle and transferred to new plates containing potato dextrose agar (PDA, Zhang et al. 2013). All putative strains of *Trichoderma* were permanently kept in the Herbarium of the Laboratory for Conservation and Utilization of Bio-resources, Yunnan University, Kunming, Yunnan, P.R. China (YMF). In addition, the holotype strains have been deposited in the China General Microbiological Culture Collection Center (CGMCC).

### Morphology characterisation and growth observation

For morphological studies, we used three different media: cornmeal dextrose agar CMD (40 g cornmeal, 2% (w/v) dextrose, 2% (w/v) agar), PDA and synthetic low nutrient agar (SNA). Each strain was first cultured on a PDA plate for 3 days and a small agar piece of 0.5 cm diam. with mycelium was then transferred respectively to new CMD, PDA and SNA plates. Strains were incubated in 9 cm diam. Petri dishes at 25 °C with a 12 h natural light and 12 h darkness interval (Sutton 1980). Colony diameters were all measured after 3 days for morphological descriptions, diameters at 25 °C and 35 °C and the times when mycelia entirely covered the surface of plate were also recorded. For microscopic morphology, photographs were taken with an Olympus BX51 microscope connected to a DP controller digital camera.

### DNA extraction, PCR amplification and sequencing

For each strain, genomic DNA was extracted from mycelium growing on PDA harvested after 3 days of growth, following the method of Wang and Zhuang (2004). For the amplifications of ITS, rpb2 and tef1 gene fragments, three different primer pairs were used: ITS4 and ITS5 for ITS (White et al. 1990), EF1-728F (Carbone and Kohn 1999) and TEF1LLErev (Jaklitsch et al. 2005) for tef1 and tRPB2-5F and tRPB2-7R for rpb2 (Chen and Zhuang 2016). Each 25 μl PCR reaction consisted of 12.5 μl T5 Super PCR Mix (containing Taq polymerase, dNTP and Mg2+, Beijing TsingKe Biotech Co., Ltd., Beijing), 1.25 μl of forward primer (10 µM), 1.25 μl of reverse primer (10 µM), 1μl DNA template, 5 μl of PCR buffer and 4.5 μl sterile water. PCR reactions were run in an Eppendorf Mastercycler following the protocols described by Zhuang and Chen (2016). PCR products were purified with the PCR product purification kit (Biocolor BioScience & Technology Co., Shanghai, China), and sequencing was carried out in both directions on an ABI 3730 XL DNA sequencer (Applied Biosystems, Foster City, California) with primers used during PCR amplification. GenBank accession numbers of sequences generated in this study are provided in Table 1.

**Table 1.** Species, strains and their corresponding GenBank accession numbers of sequences used for phylogenetic analyses.

Name	Strain	GenBank accession number		
		ITS	rpb2	tef1
*Trichodermaafarasin* P. Chaverri & Branco-Rocha	Dis 314F	FJ442259	FJ442778	FJ463400
*T.afroharzianum* P. Chaverri, F.B. Rocha & Druzhinina	GJS 04-186	FJ442265	FJ442691	FJ463301
*T.asperelloides* Samuels	GJS 04-187	JN133553	JN133560	JN133571
	GJS 04-116	GU198301	GU248411	GU248412
	GJS 08-87	–	GU198272	GU198241
*T.asperellum* Samuels, Lieckf. & Nirenberg	GJS 90-7	EU330956	EU338337	EU338333
	GJS 01-294	EU856297	FJ150788	EU856323
	GJS 06-294	GU198307	GU198266	GU198235
	CGMCC 6422	KF425754	KF425755	KF425756
	GJS 05-328	GU198318	EU248614	EU248627
*T.atrobrunneum* F.B. Rocha, P. Chaverri & Jaklitsch	GJS 04-67	FJ442273	FJ442724	FJ463360
*T.atroviride* P. Karst	DAOM 222144	AF456916	FJ442754	AF456889
*T.gamsii* Samuels & Druzhinina	GJS 04-09	DQ315459	JN133561	DQ307541
*T.guizhouense* Q.R. Li, McKenzie & Yong Wang	S628	–	KJ665273	KJ665511
*T.harzianum* Rifai	T55	KX632511	KX632568	KX632625
	T18	KX632492	KX632549	KX632606
	T2	FJ884174	KX632534	KX632591
	CBS 226.95	AY605713	AF545549	AF348101
	T11	KX632600	KX632543	KX632486
*T.hispanicum* Jaklitsch & Voglmayr	S453	JN715595	JN715600	JN715659
*T.inhamatum* Veerkamp & W. Gams	CBS 273.78	FJ442680	FJ442725	AF348099
*T.junci* Jaklitsch	CBS 120926	FJ860761	FJ860540	FJ860641
*T.kunmingense* Y. Zhang	YMF 1.02659	**KJ742800**	**KJ742801**	**KJ742802**
*T.lentiforme* P. Chaverri, Samuels & F.B. Rocha	Dis 218E	FJ442220	FJ442793	FJ463310
*T.lieckfeldtiae* Samuels	GJS 00-14	DQ109528	EU883562	EU856326
*T.lixii* P. Chaverri	GJS 97-96	AF443920	KJ665290	AF443938
*T.pleuroti* S.H. Yu & M.S. Park	CBS 124387	HM142363	HM142372	HM142382
*T.pleuroticola* S.H. Yu & M.S. Park	CBS 124383	HM142362	HM142371	HM142381
*T.pyramidale* Jaklitsch & P. Chaverri	S73	–	KJ665334	KJ665699
*T.rifaii* F.B. Rocha, P. Chaverri & Samuels	Dis 337F	FJ442621	FJ442720	FJ463321
*T.samuelsii* Jaklitsch & Voglmayr	S5	JN715596	JN715599	JN715651
*T.simmonsii* P. Chaverri, F.B. Rocha, Samuels & Jaklitsch	S7	–	KJ665337	KJ665719
*T.speciosum* Z.F. Yu & X. Du	YMF 1.00205	**MH113929**	**MH155270**	**MH183184**
*T.theobromicola* Samuels & H.C. Evans	Dis 85f	DQ109525	FJ007374	EU856321
*T.valdunense* Jaklitsch	CBS 120923	FJ860863	FJ860605	FJ860717
*T.viride* Pers	CBS 119325	DQ677655	EU711362	DQ672615
*T.yunnanense* Z.F. Yu & K.Q. Zhang	CBS 121219	GU198302	GU198274	GU198243
*T.zeloharzianum* Z.F. Yu & X. Du	YMF 1.00268	**MH113932**	**MH158996**	**MH183181**
*Nectriaeustromatica* Jaklitsch & Voglmayr	CBS 125578	HM534897	HM534887	HM534876

### Phylogenetic analyses

Preliminary BLAST searches with tef1, rpb2 and ITS gene sequences of the new isolates against NCBI and UNITE databases identified species closely related to our three isolates. Based on this information, we downloaded tef1, rpb2 and ITS sequences of 40 strains, representing 25 species. To show the phylogenetic position of *T.zeloharzianum*, 11 of the 14 species belonging to the *T.harzianum* complex were included. The remaining three species in this complex were not included because their rpb2 sequences are not available in NCBI.

Three alignment files were generated, one for each gene and converted to NEXUS files with ClustalX 1.83 (Thompson et al. 1997) to identify the phylogenetic positions of these species. The three alignments were then combined with BioEdit 7.1.9.0 (Hall 1999). The phylogenetic analyses included 1008 characters for rpb2, 1233 characters for tef1 and 590 characters for ITS. All characters were weighted equally and gaps were treated as missing characters.

Maximum Likelihood (ML) analysis was computed by RAxML (Stamatakis 2006) with the PHY files generated with ClustalX 1.83 (Thompson et al. 1997), using the GTR-GAMMA model. Maximum likelihood bootstrap proportions (MLBP) were computed with 1000 replicates. Bayesian Inference (BI) analysis was conducted with MrBayes v3.2.2 (Ronquist and Huelsenbeck 2003). The Akaike information criterion (AIC) implemented in jModelTest 2.0 (Posada and Darriba 2008) was used to select the best fit models after likelihood score calculations were done. The base tree for likelihood calculations was ML-optimised. HKY+I+G was estimated as the best-fit model under the output strategy of AIC, Metropolis-coupled Markov chain Monte Carlo (MCMCMC) searches were run for 2000000 generations, sampling every 500th generation. Two independent analyses with four chains each (one cold and three heated) were run until the average standard deviation of the split frequencies dropped below 0.01. The initial 25% of the generations of MCMC sampling were discarded as burn-in. The refinement of the phylogenetic tree was used for estimating Bayesian inference posterior probability (BIPP) values. The Tree was viewed in FigTree v1.4 (Rambaut 2012), values of Maximum likelihood bootstrap proportions (MLBP) greater than 70% and Bayesian inference posterior probabilities (BIPP) greater than 90% at the nodes are shown along branches.

## Results

### Sequence analyses

The final alignments and the trees obtained have been deposited in TreeBASE (TreeBASE accession number: 23172). Phylogenetic positions of the new species were ascertained by analyses of the combined tef1, rpb2 and ITS dataset containing 2831 characters, of which 487 characters were constant, 2344 were variable.

In our analyses, sequences from 41 strains including 21 strains of the Harzianum Clade, 19 strains of the Viride Clade and an outgroup taxa, *Nectriaeustromatica* were used to construct the phylogenetic tree. Of the three new species, *T.speciosum* and *T.kunmingense* belonged to the Viride Clade, whereas *T.zeloharzianum* were located in the Harzianum Clade. These two clades formed a monophyletic group, which is generally consistent with what was found in a previous study (Jaklitsch and Voglmayr 2015). The three new species each clustered with different species to form well-supported clades. *T.speciosum* was closely related with *T.samuelsii* Jaklitsch & Voglmayr, *T.hispanicum* (Jaklitsch & Voglmayr) Jaklitsch & Voglmayr and *T.junci* Jaklitsch. This clade had high statistics support (BIPP/MLBP = 100%/85%). *T.kunmingense* fell within a clade formed by strains of *T.asperellum* Samuels, Lieckf. & Nirenberg, but there was a distinct genetic distance between *T.kunmingense* and strains of *T.asperellum*. Similarly, *T.zeloharzianum* was phylogenetically distinct but associated with *T.harzianum* Rifai, *T.lixii* (Pat.) P. Chaverri and *T.simmonsii*. Jaklitsch & Voglmayr.

**Figure 1.**
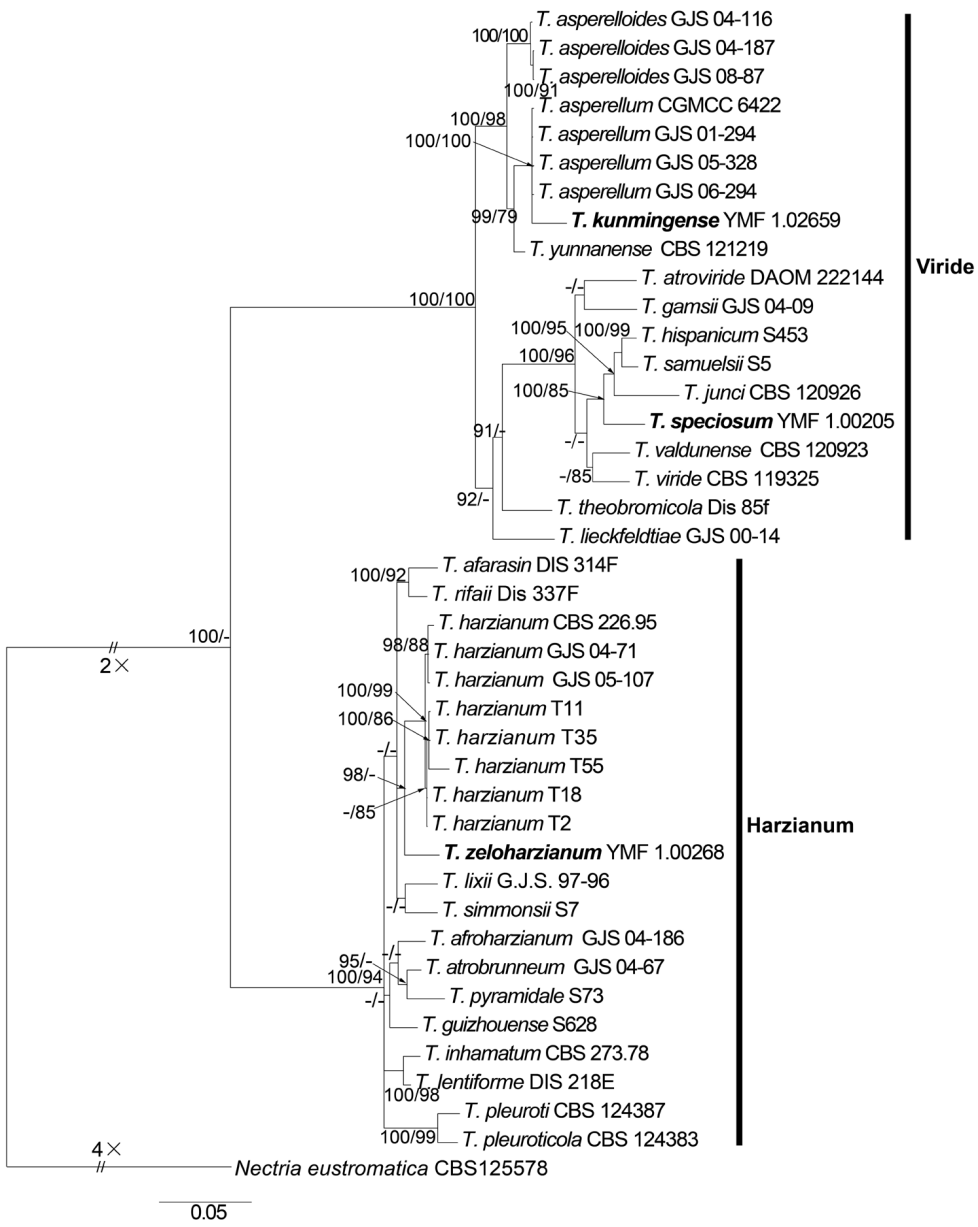
Phylogenetic tree based on Bayesian analysis of the combined tef1, rpb2 and ITS sequences. *Nectriaeustromatica* is used as the outgroup. Bayesian posterior probabilities greater than 0.90 are given at the nodes (left). Maximum likelihood bootstrap values greater than 70% are given at the nodes (right). The scale bar shows the expected changes per site. New species proposed are in boldface.

### Taxonomy

#### Trichoderma speciosum

Taxon classificationFungiHypocrealesHypocreaceae

Z.F. Yu & X. Du sp. nov.

825469

Figure 2

##### Etymology.

**Latin**, *speciosum* refers to showy and splendid colony on PDA.

##### Diagnosis.

Characterised by tree-like conidiophores, branches paired or in whorls of 3–4, spindly to fusiform phialides (5.0–10.0 × 2.0–3.0 μm), subglobose to globose conidia (3.7–4.9 × 3.1–3.8 μm). Differs from *T.hispanicum* by paired branches, whorled and thinner phialides, subglobose to globose conidia. Differs from *T.samuelsii* by paired and compact branches, subglobose to globose conidia and the character of pustules on SNA. Differs from *T.junci* by whorled, smaller phialides and subglobose to globose conidia.

**Figure 2.**
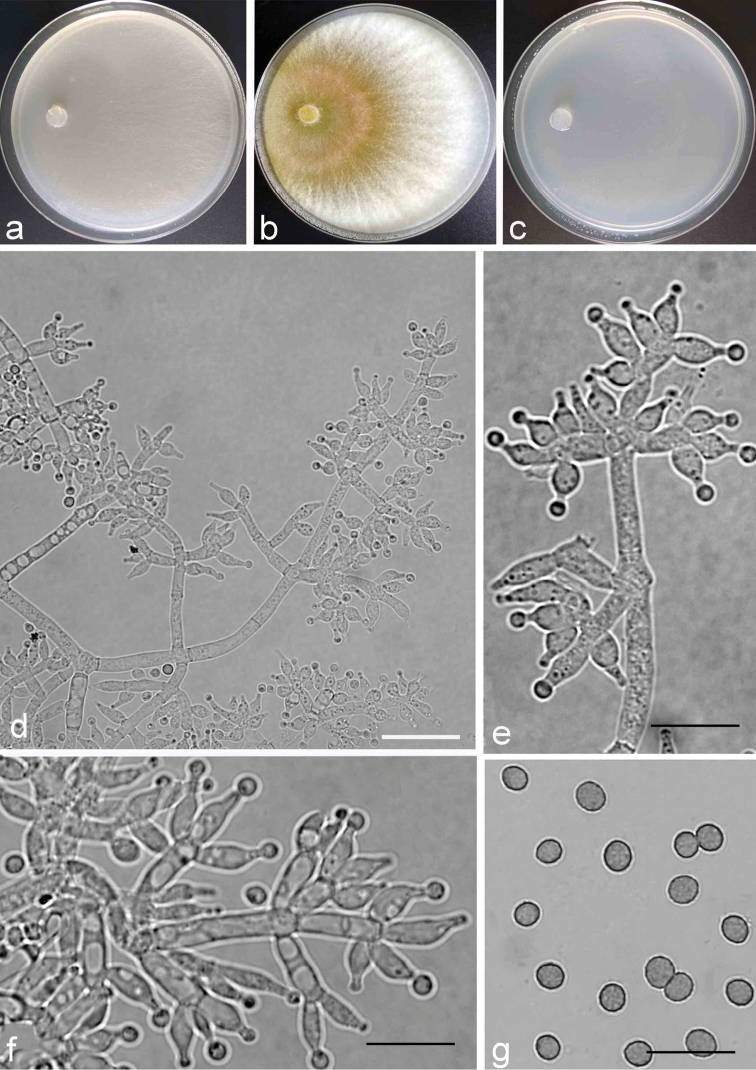
Cultures and anamorph of *Trichodermaspeciosum*. **a–c** Cultures (**a** on CMD, 3 days **b** on PDA, 3 days **c** on SNA, 3 days) at 25 °C **d–f** Conidiophores and phialides (SNA, 4 d) **g** Conidia (SNA, 20 d); Scale bars: 10 μm (**d–g**).

##### Type.

**CHINA.** From soil of tobacco rhizosphere, Luliang, Yunnan Province, 24°57'22"N, 103°46'30"E, 1800 m alt., Jul 2007, Z.F. Yu (YMF 1.00205, holotype), Ex-type culture CGMCC 3.19079.

##### Description.

Mycelium covers plate after 72 h at 25 °C and 30 °C on CMD, no growth at 35 °C. Colony homogenous, pale yellowing, not zonate, outline circular. Aerial hyphae sparse, relatively abundant at margin, distinctly radial, arachnoid. Conidial production noted after 4 days.

On PDA, mycelium covers the plate after 72 h at 25 °C and 30 °C, no growth at 35 °C. Colony circular, typically zonate, yellow-green colony homogeneous distributed around the point of inoculation, forming a coarse circle. Whitish aerial hyphae distributed on the agar surface in external zone, hairy, dense and radial. Conidial production noted after 3 days.

On SNA after 72 h, colony radius 37–38 mm at 25 °C, mycelium covers the plate after 120 h, 56–59 mm at 30 °C after 72 h, no growth at 35 °C. Colony hyaline, thin, fan-shaped with indistinct outline. Aerial hyphae scarcely degenerating. Conidial production noted after 5 days, minute white pustules formed around central part of the colony, turning green after 6 days. Conidiophores tree-like, comprising a main axis with second branches, base 3.0–4.0 μm wide, second branches paired or in whorls of 3, sometimes second branches branched again, the distance between neighbouring second branches is (12.0–) 15.0–29.0 (–30.0) μm, main axis and branches terminating in whorls of up to five phialides. Conidiogenous cells phialides lageniform or ampulliform, arising singly or in 2–4; 5.0–10.0 × 2.0–3.0(–3.5) μm, length/width ratio 1.7–3.6 (–4.2), non-equilateral when curved. Conidia ovoid to short ellipsoidal, verrucose (3.6–)3.7–4.9(–5.0) × (3.0–)3.1–3.8(–4.2) μm, length/width ratio (1.0)1.1–1.4(–1.5).

##### Habitat and distribution.

In soil from tobacco rhizosphere in part of cultivated land of south-western China.

##### Teleomorph.

**Not known**

##### Remarks.

*Trichodermaspeciosum* is phylogenetically most closed related to three species – *T.hispanicum*, *T.samuelsii* and *T.junci* (Jaklitsch et al. 2012; Jaklitsch 2011). The three species were isolated from ascospores and only *T.speciosum* was isolated from the anamorph. However, *T.speciosum* differs from these three species in having verrucose, subglobose to globose conida, while conidia of *T.hispanicum* and *T.samuelsii* are oblong and smooth and those of *T.junci* are ovoid to ellipsoidal with length/width ratio 1.3–1.8(–2.2).

In addition, side branches of *T.hispanicum* are often unpaired, phialides often singly, whereas branches of *T.speciosum* are generally paired or in whorls of 3–5. For *T.samuelsii*, branches are sparser and phialides with l/w of (1.7–)2.5–4.6(–7.1) are more slender than those of *T.speciosum*. Phialides of *T.junci* are also more slender than those of *T.speciosum*, which are narrowly lageniform.

#### Trichoderma kunmingense

Taxon classificationFungiHypocrealesHypocreaceae

Z.F.Yu & J.Y.Li sp. nov.

808878

Figure 3

##### Etymology.

**Latin**, *kunmingense*, refers to the site in which this species was found.

##### Diagnosis.

Characterised by pyramidal fashion conidiophores, ampulliform to tapered phialides (6.0–9.0 × 2.5–4.5 µm), discrete branches and ovoid, occasionally ellipsoid, smooth-walled conidia (3.4–4.4 × 2.7–3.4 µm). Differs from *T.asperellum* by slightly shorter and sometimes more whorled phialides, mostly obovoid conidia. Differs from *T.yunnanense* by sparser branches and more whorled, smaller phialides and conidia.

##### Type.

**CHIN**A. Kunming, Yunnan, 24°52'28"N, 102°49'34"E. 1929 m alt, in soil, Aug 2007, Y. Zhang (YMF 1.02659, holotype), Ex-type culture CBS 125635.

**Figure 3.**
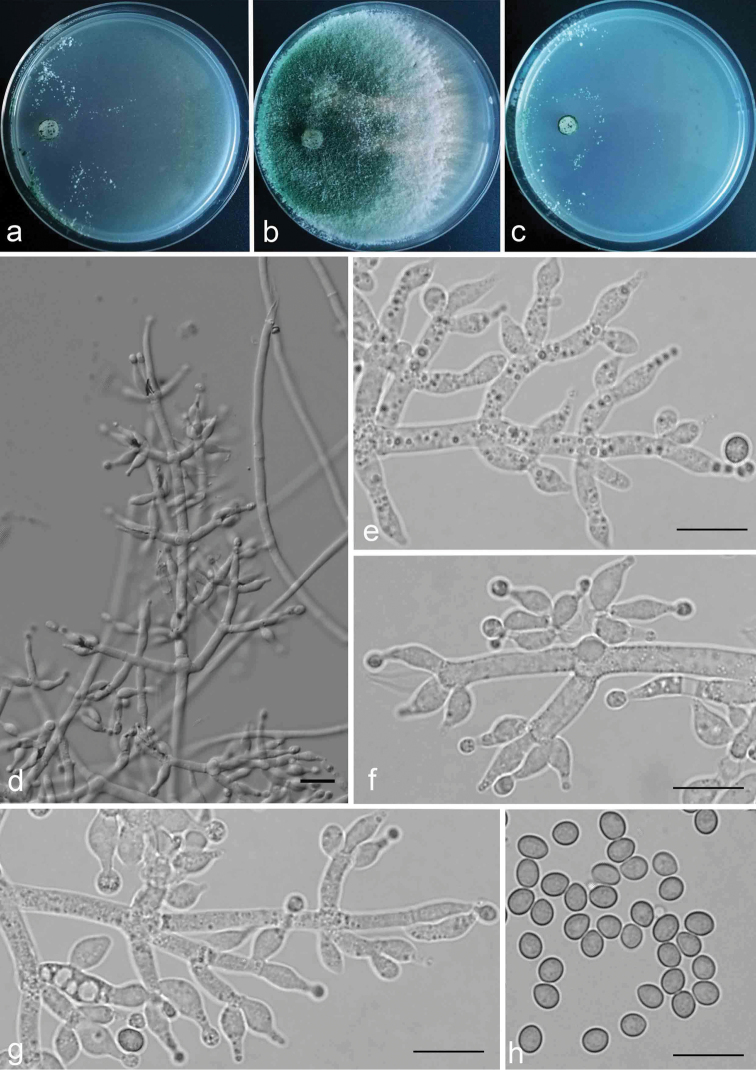
Cultures and anamorph of *Trichodermakunmingense*. **a** on CMD at 30 °C, 3 days **b, c** Cultures (**b** on PDA, 3 days **c** on SNA, 3 days) at 25 °C **d–g** Conidiophores and phialides (SNA, 4 d) **h** Conidia (SNA, 20 d); Scale bars: 10 μm for (**d–h**).

##### Description.

Colony on CMD after 72 h radius 35–50 mm, mycelium covering the plate after 96 h at 25 °C, 55–59 mm at 30 °C and 41–46 mm at 35 °C after 72 h. Colony hyaline, margin distinctly noted. Aerial hyphae are indistinctly observed, radiate and sparse, white pustule formed from inner zone, asymmetrical to pulvinate, loosely arranged. Conidial production noted after 48 h. No diffusing pigment produced.

Mycelium covers plate after 72 h at 25 °C and 35 °C on PDA and radius 52–56 mm at 30 °C. Colony layered distinctly, margin conspicuous and radial. Aerial hyphae, hairy to floccose, dense internal zone, but relative sparse on margin, abundantly and flat in a large green disc around the inoculums, turning green after 24 h of conidiation.

Colony on SNA after 72 h radius 48–50 mm, mycelium covering the plate after 96 h at 25 °C, 53–56 mm at 35 °C and covering the plate at 30 °C after 72 h. Colony and pustules are similar to that on CMD, colony hyaline and smooth, the shape of pustules more regular, sometimes hemispherical, loosely distributed around the point of inoculation. Conidiophores well defined, branching 2–3 times in a pyramidal fashion, with the longest branches verticillate on the discrete main axis, the base 2.2–3.9(–4.4) μm wide, branched toward the tip, the distance between neighbouring second branches are 11.0–38.5 μm. Phialides arising generally 1–3 times repetition on each branches or in whorls of 3–5, ampulliform to tapered, slightly constricted at the base, often straight or less sinuous or curved toward apex of conidiophore, mostly (5.0–) 6.0–9.0(–10.0) × 2.5–4.5 µm, length/width ratio (1.3–)1.4–3.4(–3.6). Conidia obovoid, sometimes ellipsoidal, smooth-walled, both ends broadly rounded or at the base slightly narrower, 3.4–4.4 × 2.7–3.4 µm, length/width ratio (1.1–)1.2–1.6, pale green when viewed singly, usually greenish in mass.

*Specimen examined.* PR China, Kunming, Yunnan Province, 24°52'N, 102°49'E, elev. 1929 m, isolated from soil samples, Aug. 2007, by Y. Zhang (Holotype, YMF 1.02659; ex-type culture, YMF 1.026591, CBS 125635).

##### Habitat and distribution.

In garden soil of Kunming city of southwest China.

##### Teleomorph.

**Not known**

##### Remarks.

*Trichodermakunmingense* can be distinguished from *T.asperellum* Samuels, Lieckfeldt and Nirenberg, by having more crowded branches and phialides. *T.asperellum* typically forms whorls of 2–4 phialides, whereas phialides of *T.kunmingense* sometimes attain 5 phialides. Although the phialides are ampulliform in both species, the phialides of *T.asperellum* are slightly longer (type strain: 7.2–11.5 µm) than those of *T.kunmingense*. Moreover, conidia of *T.asperellum* have inconspicuous and small ornamentation, but those of *T.kunmingense* are smooth and conidia are slightly longer (type strain: 3.5–4.5 × 2.7–4.0 µm) (Samuels et al. 1999, Samuels and Ismaiel 2010).

*Trichodermakunmingense* and *T.yunnanense* Yu and Zhang are also closely related in the phylogenetic tree, but branches and phialides of *T.yunnanense* are more crowded than those of *T.kunmingense*. Phialides in *T.yunnanense* arising separately or more often paired with branches, rarely in whorls of 3 (Yu et al. 2007). Conidia of *T.yunnanense* (4.0–5.0 ×3.5–4.0 µm) are also larger than those of *T.kunmingense*.

#### Trichoderma zeloharzianum

Taxon classificationFungiHypocrealesHypocreaceae

Z.F. Yu & X. Du sp. nov.

825472

Figure 4

##### Etymology.

Greek *zelo*-, meaning emulation + *harzianum*, referred to *Trichodermaharzianum*

##### Diagnosis.

Characterised by pyramidal conidiophores, verticillate branches, ampulliform to lageniform phialides (5.5–10.0 × 2.5–3.5 μm) and subglobose to globose, thin-walled conidia (2.7–3.1 × 2.4–2.6 μm). Differs from *T.harzianum* by verticillate branches, 3–6 whorled phialides on terminal of each branch and thinner conidia. Differs from *T.lixii* by verticillate and compact branches, more terminal phialides on main axis and smaller conidia. Differs from *T.simmonsii* by verticillate branches and longer conidia.

**Figure 4.**
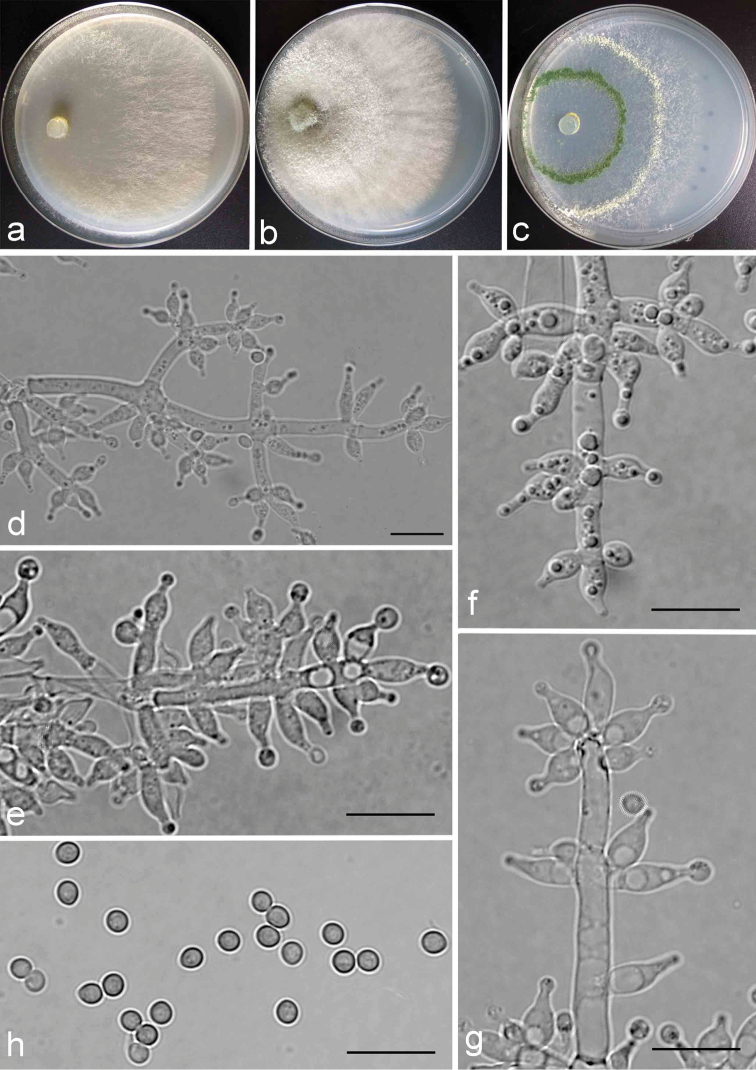
Cultures and anamorph of *Trichodermazeloharzianum*. **a–c** Cultures (**a** on CMD, 3 days **b** on PDA, 3 days **c** on SNA, 3 days) at 25 °C **d** Conidiophore-like structures (SNA, 4 d) **e–g** Conidiophores and phialides (SNA, 4 d) **h** Conidia (SNA, 20 d); Scale bars: 10 μm for **d–h**.

##### Type.

**CHINA.** Yunnan: Qujing City, Luliang county, 25°05'25"N, 103°56'42"E, 1800 m alt., in soil, Jul 2007, Z.F. Yu (YMF 1.00268, holotype), Ex-type culture CGMCC 3.19082.

##### Description.

On CMD after 72 h, colony radius 59–62 mm, mycelium covers the plate after 96 h at 25 °C; 43–45 mm at 30 °C and 46–52 mm at 35 °C after 72 h. Colony yellowing, margin distinct. Aerial hyphae fertile and conspicuous, hairy radial, distributed on surface, green conidial production noted after 4 days.

On PDA after 72 h, colony radius 57–58 mm, mycelium covers the plate after 96 h at 25 °C. Covering the plate at 30 °C and 38–42 mm at 35 °C after 72 h. Colony white, margin distinct. Aerial hyphae abundant, hairy to floccose, denser around central disc. Green conidiation noted after 3 days.

On SNA after 72 h, radius 59–65 mm, mycelium covers the plate after 144 h at 25 °C, 64–65 mm at 30 °C and 29–37 mm at 35 °C after 72 h. Aerial hyphae sparsely, slightly radial and conspicuous zonate. Conidiophores well defined, branching 2–3 times in a pyramidal fashion. Branches paired or a whorl of 3–4, the distance between neighbouring second branches is 16.0–39.0 μm, base 3.0–4.0 μm wide, each branch terminating in a whorl of 3–6 phialides, phialides ampulliform to lageniform, often verticillated up to 5 around the main axis near the apex, rarely singly arising, (4.5)5.5–10.0(–11.0) × 2.5–3.5(–4.0) μm, length/width ratio (1.4–)1.8–3.4(–3.6). Conidia smooth on surface, subglobose to globose, sometimes obovoid, (2.6–) 2.7–3.1(–3.2) × (2.3–) 2.4–2.6(–2.7) μm, length/width ratio (1.0–)1.1–1.3(–1.4).

##### Habitat and distribution.

In soil from tobacco rhizosphere in part of cultivated land of south-western China.

##### Teleomorph.

**Not known**

##### Remarks.

*Trichodermazeloharzianum* forms a single branch with *T.harzianum* Rifai as sister clade. Morphologically, *T.harzianum* is similar to *T.zeloharzianum* in their shape of conidiophores and phialides, but the branches of *T.harzianum* are opposite of each other and each branch terminating in a whorl of 2–5 phialides (Chaverri et al.2015), while *T.zeloharzianum* is clearly distinguishable by having verticillated branches and 3–6 terminal whorled phialides. In addition, the conidia of *T.harzianum* are generally wider [(2.0−)2.5−3.0 (−3.7) μm] than those of *T.zeloharzianum*.

*Trichodermalixii* differs from *T.zeloharzianum* also by having opposing pairs of branches and fewer terminal phialides (2–4) on main axis. Beyond that, closely spaced branches are common in *T.lixii* (Chaverri et al.2015), whereas for *T.zeloharzianum*, neighbouring branches are more compact and the conidia of *T.lixii* are usually larger [(2.5−)3.0−3.5 (−3.7) × (2.2−)2.5−3.2(−3.5) μm] than those of *T.zeloharzianum*.

*Trichodermasimmonsii* is also distinguished obviously from *T.zeloharzianum*, except their differences about opposing branches (Chaverri et al. 2015), the phialides are more stout and shorter ((4.2−)5.2−6.5 (−9.0) μm) than those of *T.zeloharzianum*. Furthermore, *T.simmonsii* is commonly constricted below the tip to form a narrow neck (Chaverri et al. 2015); however, this character is not found in *T.zeloharzianum*.

## Discussion

The application of molecular barcode for fungal taxonomy has led to a re-evaluation of morphology-based taxonomy of *Trichoderma*. A recent study suggested that tef1 introns could provide a high resolution to this genus and is shown to be superior to other phylogenetic markers (Jaklitsch et al. 2012). Rpb2 sequences appeared powerful due to their suitable interspecific variations (Jaklitsch and Voglmayr 2015). ITS sequences are identical or nearly identical for several species of the genus (e.g. those of *T.hispanicum*, *T.koningii*, *T.viridescens* and *T.samuelsii*), therefore this marker alone is not useful for phylogenetic reconstruction or for barcoding of these fungi (Druzhinina et al. 2005, Jaklitsch et al. 2012). Together, due to their universality and reliability for species in the *Trichoderma* genus, these three loci were chosen for this study.

Based on the combined analysis of sequences from three genes, phylogenetic positions of three species were ascertained, amongst which *T.zeloharzianum* belonged to the Harzianum clade. *T.zeloharzianum* has the characteristic of typical *T.harzianum*-like morphology containing pairs or verticils branches, ampulliform to lageniform phialides and globose to subglobose or broadly ovoid conidia (Chaverri et al. 2015). The *T.harzianum* species complex is a cosmopolitan and ubiquitous species, playing important roles in ecology and economy. Chaverri et al. (2015) disentangled this species complex recognising 14 species. In the present study, 11 of the 14 species from the Harzianum Clade were included for analyses. *T.zeloharzianum* is the most closely related to *T.harzianum*, with the latter being more broadly distributed. The sexual and asexual morphs for *T.lixii*–*T.harzianum* have been rejected (Druzhinina et al. 2010, Atanasova et al. 2013) and Chaverri et al. (2015) and also showed that *T.lixii* and *T.harzianum* are closely related but represent separate species. Here, we found *T.zeloharzianum* is more closely to *T.harzianum* than to *T.lixii*.

Both *T.speciosum* and *T.kunmingense* belong to the Viride Clade. The study of Jaklitsch and Voglmayr (2015) indicated that the structure of the Viride Clade is complex, as there are additional subclades, such as the Hamatum/ Asperellum Clade, the Rogersonii Clade, the Neorufum Clade and several smaller subclades. Samuels et al. (2006)showed that asexual morphs of the Viride Clade often have verrucose conidia. In the present study, *T.kunmingense* with smooth conidia is found phylogenetically related to *T.asperellum* and *T.asperelloides*, two species with verrucose conidia and both belonging to the Asperellum subclade. However, *T.speciosum* with warted conidia could not be assigned to any specific subclade.

Species of the Harzianum and Viride Clades were commonly isolated from soil. However, the number of published soil-inhabiting *Trichoderma* species is limited compared with that on woody substrates. Furthermore, the sexual states of most soil-inhabiting species are unknown (Chen and Zhuang 2016). China is rich in species diversity of the *Trichoderma* genus. Future studies will likely reveal more new taxa in soil, which could provide a better understanding of the relationship between asexual and sexual states of some species in the genus.

## Supplementary Material

XML Treatment for Trichoderma speciosum

XML Treatment for Trichoderma kunmingense

XML Treatment for Trichoderma zeloharzianum
